# Overcoming barriers to seedling regeneration during forest restoration on tropical pasture land and the potential value of woody weeds

**DOI:** 10.3389/fpls.2014.00200

**Published:** 2014-05-20

**Authors:** Amelia T. Elgar, Kylie Freebody, Catherine L. Pohlman, Luke P. Shoo, Carla P. Catterall

**Affiliations:** ^1^School of Environment, Environmental Futures Research Institute, Griffith UniversityBrisbane, QLD, Australia; ^2^Tablelands Community Revegetation UnitMalanda, QLD, Australia; ^3^Centre for Rainforest Studies, The School for Field StudiesYungaburra, QLD, Australia; ^4^School of Biological Sciences, The University of QueenslandSt Lucia, QLD, Australia

**Keywords:** rainforest, regrowth, seed dispersal, novel ecosystem, old field, plant invasion

## Abstract

Combating the legacy of deforestation on tropical biodiversity requires the conversion to forest of large areas of established pasture, where barriers to native plant regeneration include competition with pasture grasses and poor propagule supply (seed availability). In addition, initial woody plants that colonise pasture are often invasive, non-native species whose ecological roles and management in the context of forest regeneration are contested. In a restoration experiment at two 0.64 ha sites we quantified the response of native woody vegetation recruitment to (1) release from competition with introduced pasture grasses, and (2) local facilitation of frugivore-assisted seed dispersal provided by scattered woody plants and artificial bird perches. Herbicide pasture grass suppression during 20 months caused a significant but modest increase in density of native woody seedlings, together with abundant co-recruitment of the prominent non-native pioneer wild tobacco (*Solanum mauritianum*). Recruitment of native species was further enhanced by local structure in herbicide-treated areas, being consistently greater under live trees and dead non-native shrubs (herbicide-treated) than in open areas, and intermediate under bird perches. Native seedling recruitment comprised 28 species across 0.25 ha sampled but was dominated by two rainforest pioneers (*Homalanthus novoguineensis*, *Polyscias murrayi*). These early results are consistent with the expected increase in woody vegetation recruitment in response to release from competitive and dispersive barriers to rainforest regeneration. The findings highlight the need for a pragmatic consideration of the ecological roles of woody weeds and the potential roles of “new forests” more broadly in accelerating succession of humid tropical forest across large areas of retired agricultural land.

## Introduction

Approximately half of the world's tropical biomes have been subjected to some form of clearing (Asner et al., 2009). One of the major drivers behind deforestation of tropical forests is clearing for agricultural practices (Achard et al., 2002). Consequently, large tracts of continuous rainforest have been converted to fragmented patches of remnant forest and secondary regrowth, situated amongst mosaics of agricultural land and cattle pastures (Turner and Corlett, 1996). This process threatens global biodiversity, and causes increased global carbon emissions and changes in ecosystem functioning (Bradshaw et al., 2008). In some tropical forest landscapes, areas that were initially cleared for pasture and cattle grazing are eventually abandoned, due to declining productivity of pasture grasses, ongoing soil degradation, invasion of unpalatable grasses and changing socio-economic incentives (Hobbs and Cramer, 2007; Grau and Aide, 2008).

Forest recovery may subsequently occur in these retired tropical pastures, but several ecological factors act to reduce colonization by rainforest plants, potentially leaving the landscape in a state of arrested succession (Holl et al., 2000; Kanowski et al., 2009). Competition plays an important role in these dynamics, because a persistent cover of pasture grasses and herbs can limit forest regeneration following the removal of grazing livestock, by restricting micro-climatic conditions required for seed germination and the access of newly recruited woody seedlings to light, soil moisture or nutrients (Holl, 2002). Additionally, recovery of tropical forests is also often limited by a lack of propagule (seed) supply, because the seeds of many rainforest tree and shrub species have short-duration viability and are quickly exhausted from the soil seed bank during prolonged land use (Uhl, 1987; Holl et al., 2000). Furthermore, they are typically produced within fleshy fruits, so that their seed dispersal is mediated by frugivorous vertebrates that do not frequently visit open pasture (Da Silva et al., 1996; Wunderle, 1997).

Rapid reforestation over areas that are sufficiently large to be ecologically useful requires management interventions to reduce these barriers to regeneration. A variety of such interventions have been explored in recent years by an emerging cohort of restoration practitioners (Shoo and Catterall, 2013). For example, suppression of pasture grasses through various methods can in some circumstances promote reestablishment of woody vegetation (Shoo and Catterall, 2013). Additionally, the presence of isolated paddock trees potentially attracts seed dispersing animals which may move from forest to pasture and thereby facilitate seedling establishment (Guevara et al., 2004; Manning et al., 2006). Installation of artificial perches may similarly encourage seed rain, although subsequent seedling recruitment appears limited when pasture grasses are present (Holl, 1998; Shiels and Walker, 2003; Graham and Page, 2012). By contrast, canopy shade provided by live established paddock trees could competitively limit the growth of pasture grasses and herbs as well as potentially improving micro-climatic and soil conditions toward a more favorable environment for rainforest seedling recruitment (Rhoades et al., 1998; Manning et al., 2006). However, for these and other potential management interventions, there has been only limited systematic assessment of their effectiveness (Shoo and Catterall, 2013). A further dimension of management complexity occurs because non-native invasive plants are often the first and most abundant woody species recruited into retired pasture; these could act as recruitment facilitators for native forest seedlings by both attracting frugivores and shading the ground, but their treatment in restoration is controversial because of the possibility that they may also have negative effects on forest recruitment (D'Antonio and Meyerson, 2002; Kanowski et al., 2008; Davis et al., 2011).

Here we investigate methods for encouraging recruitment of rainforest seedlings in retired tropical pasture by removing or manipulating certain ecological barriers. We use a realistically scaled management experiment established in the Wet Tropics uplands of north eastern Australia to quantify the short term response of native woody seedling recruitment to: (1) herbicide-induced release from competition with introduced pasture grasses; and (2) local facilitation provided by elements of habitat structure, specifically scattered trees, shrubs, and artificial bird perches. We test whether rainforest seedling recruitment increases when competitive and dispersive barriers to regeneration are reduced. We also describe the extent and pattern of co-recruitment by non-native invasive woody plants, and discuss the findings in the context of current understanding of old field restoration in the tropics, with a particular emphasis on the contentious role of woody weeds in efforts to reinstate forest over large areas.

## Methods

### Study area

An experimental restoration project (“Kickstart Pasture Conversion Trials”) was established in November 2011 in areas adjoining the Mt Hypipamee/Upper Barron section of the Wet Tropics World Heritage Area, on the Atherton Tableland, north eastern Australia. The native vegetation is complex notophyll to mesophyll vine forest (Tracey, 1982). Landscape vegetation cover at the time of this study was a mosaic of remnant forest, substantial areas of livestock pasture (from which forest was cleared mainly in the first 5–6 decades of the twentieth century), and regrowth forest of varying ages. Pasture areas were mainly used for cattle grazing, and by the end of the century were dominated by non-native tropical grasses, such as signal grass (*Urochloa decumbens*) and pasture legumes, with guinea grass (*Megathyrsus maximus*) and setaria (*Setaria sphacelata* var. *anceps*) also widely established, together with a wide variety of other planted and invasive species.

So far, three kickstart pasture conversion sites have been established, each on red basaltic soil in an area of retired pasture which slopes steeply northwards to an east-flowing gully bordering a large area of conserved rainforest. In this paper we restrict most analyses to two sites from which the longest data time series is available (CloudlandE and CloudlandW; Figure S1), located on a single property (Cloudland: 17° 27′ 59″ S, 145° 32′ 28″ E, 875 m elevation), and separated by approximately 400 m which includes a strip (150 m wide) of restored rainforest that was planted in 2007. Limited shorter-term data are also presented from the third, later-established, site (Ringtail: 17° 27′ 59″ S, 145° 32′ 28″ E, 821 m elevation, some 3 km from Cloudland). The Cloudland property experienced cycles of partial clearing and regrowth since before the 1940s, and was partly cleared and grazed in the 1980s and 1990s, until 2005 when the entire property was destocked followed by ongoing exclusion of all grazing livestock. At the commencement of the study, pasture grass cover remained dense and was taller (often 0.5–1.0 m) than when grazed, and was variably intermixed with scattered colonising woody plants 2–8 m tall, comprising either native rainforest or non-native invasive species, together with occasional other small patches of low (<1.5 m tall) shrub or vine growth. Nearby weather stations indicate an average annual rainfall of 1467 mm, with 75% of the total falling between December and April (2001–2013, 2.6 km away at 031184 McKell Road Alert) and average monthly minimum and maximum temperatures of 14.7 and 25.4°C respectively (1961–1990, 16.5 km away at 031029 Herberton Mowbray Rd).

### Design of experimental restoration treatments

Each site contained two types of plot 8–200 m apart within the retired pasture, each plot being a square 80 m by 80 m (0.64 ha) with one side abutting the rainforest edge: (1) the “Works plot” within which all experimental management works took place; and (2) the “Control plot,” a similar delineated area of retired pasture which remained untreated (Figures S1, S2). Management interventions in each Works plot were of two types: (1) grass and herb suppression; and (2) installation of bird-attracting structures. In addition, we also investigated (3) the influence of pre-existing trees and shrubs.

Grasses, herbs and other low-growing pasture-associated plants were suppressed with repeated herbicide applications, at a frequency which depended on weather conditions and observed grass or herb regrowth, as judged during regular site inspections (in general, grass and herb growth was slower during the cooler winter months). To reduce external influences, a 5.0 m wide buffer around the perimeter of the Works plot was also treated with herbicide. Herbicides were either glyphosate (which has a broad spectrum action on all types of plant) or the grass-selective Fusilade (fluazifop-p butyl) and Verdict (haloxyfop-R-methyl). The initial herbicide treatment in each Works plot aimed to achieve comprehensive glyphosate coverage of all ground-level plants, with no attempts to locate or protect small native forest seedlings, very few of which were apparent. Subsequent treatments were either spatially selective sprays with glyphosate, to spare any recruiting native rainforest seedlings, or more generalized sprays of grass-selective herbicide. Concentrations and application methods were those typically used by experienced restoration practitioners during establishment and maintenance of rainforest replanting projects in the region. Decisions about the type and delivery of herbicide (e.g., application timing; broad-spectrum vs. grass-selective chemicals; broadcast or localized delivery; use of vehicle-based high-pressure spray or backpack) were made independently and progressively for each site, depending on local topography, how the vegetation had recently developed, and the type of herbicide involved. These decisions were also guided by the underlying goal of longer term cost efficiency within a management context. Overall, during 20 months there were 7–10 herbicide treatments (varying in different parts of the plot) at CloudlandE, and seven at CloudlandW; and during the 9 months at Ringtail there were 6–7 sprays. Some treatments involved broadscale grass suppression, while others were more spatially localized, and targeted specific clumps of herbs, grasses, shrubs or scrambling vines. The nature of these treatments requires close tailoring of actions to suit each local combination of climate and ground-cover species.

Each bird attracting structure (henceforth a “perch”) was constructed from an existing multi-branched *Alphitonia petriei* (a common early successional tree), cut at the base, with branches pruned back to a standard form (resulting height about 3–4 m, diameter 5–10 cm with 3–5 branches, embedded in the ground to a depth of 0.5 m). At the base of each sapling there was a water tray (surface 38 × 25 cm, depth 15 cm) which filled mainly from ambient rainfall with occasional hand supplementation, together with two logs (each about 1.0 m long and 20–25 cm diameter). Nine perches were installed in each Works plot in a regular grid of 20 m spacing, with three rows of three perches at each of 20, 40, and 60 m away from, and parallel to, the forest edge.

The pre-existing trees and shrubs in each plot were mapped if they were >2.0 m tall. These consisted of two types: (1) small native regrowth trees (henceforth termed “live trees”), and (2) non-native woody shrubs, all of which were treated with herbicide as part of the initial Works plot establishment (henceforth termed “dead clumps”). Live trees were mostly *Alphitonia petriei*, *Acacia celsa*, *Rhodamnia sessiliflora*, and *Xanthophyllum octandrum*. Care was taken during herbicide applications to avoid these trees. Dead clumps were almost all either lantana (*Lantana camara*) or wild tobacco (*Solanum mauritianum*), and were killed by stem treatment with Starane (fluroxypyr). A few small localized patches of very low growing native shrub (e.g., *Rubus queenslandicus*) or other small woody non-native plants were also killed with herbicide treatment during initial plot establishment.

### Field data collection

Here we present data from seedling recruitment surveys conducted 20 months after the Works plots were established at the two Cloudland sites (i.e., in July 2013), and 9 months after the establishment of the Ringtail Works plot. Seedling recruitment was measured in two types of systematic search area: strips and circles. Strips were positioned regularly throughout both the Works and Control plots, and circles were placed around perches, live trees or dead clumps. In both types of search, all recruits of woody-stemmed trees or shrubs were counted and identified to species level if they: (1) were seedlings (i.e., excluding a few non-native shrubs that re-sprouted from rootstock); (2) were >10 cm in height; and (3) belonged to any species (native or non-native) which develops a woody stem that can typically exceed 2.5 cm diameter as individuals grow. The height of each stem was also measured, up to a maximum of 2.0 m (after which diameter was recorded).

Strips were transects 20 m by 2 m, positioned parallel to the forest edge. An arrangement of four strips laid end to end spanned the full width of the plot, and this was repeated at eight distances from the forest edge (5, 15, 25, 35, 45, 55, 65, 75 m), giving a total of 32 search strips in each plot (Figure S2). Circles had a radius of 2 m, which was placed around either the main stem of tree life-forms or a marked approximate center point within a multi-stemmed clump (the latter being typical for lantana). The number of circles surveyed in a plot depended on how many live trees, dead clumps and perches were present; in the Works plot at CloudlandE these numbers were 7 live trees, 0 dead clumps and 9 perches; compared with 5, 10 and 9 respectively at CloudlandW, and 11, 3, and 9 respectively at Ringtail.

### Data analyses

Counts of recruited seedlings in strips and circles (see above) were standardized to a density unit of stems per 100 m^2^ to account for the difference in sampling area between search types (i.e., 40 m^2^ for strips and 12.6 m^2^ for circles). To examine the effect of grass suppression, mean recruit densities were compared between Works and Control plots, with strips as replicates but excluding all strips that intersected with live trees or dead shrubs, to remove the potential confounding effect of structure on recruitment (no strips intersected with circles around perches; Figure S2). Resulting sample sizes were 25 and 26 strips for Works and Control plots respectively at CloudlandE, 21 and 22 respectively at CloudlandW, and 23 and 6 respectively at Ringtail. To test the effect of structural features when acting in combination with grass suppression, we restricted data analysis to the herbicide-treated Works plots and compared mean recruit densities among four contexts: open areas (i.e., strips that did not intersect perches, live trees or dead shrubs) and circles beneath each of these three types of structure. In each case, separate analyses were conducted for native species collectively and non-native species collectively.

Significance tests of treatment effects were conducted using data from the two Cloudland sites, for which 20 month data were available. Tests were performed using generalized linear models based on a Poisson error structure and log link function, implemented using the glm function in the base “stats” library of R (version 3.0.2; R Core Team, 2013). Each model contained three types of fixed effect: restoration treatment (i.e., grass suppression or types of structure as described above), site (CloudlandE or CloudlandW) and their interaction. We evaluated the significance of each term by comparing full models that included all terms and interactions with reduced models omitting each term in turn, using chi-squared tests on the differences in explained variance (Faraway, 2006). Finally, Spearman's correlations were performed to test for an association between numbers of native seedlings recruited and area-specific estimates of species richness for strip and circle searches.

## Results

### Extent of seedling recruitment

**Figure 1:**
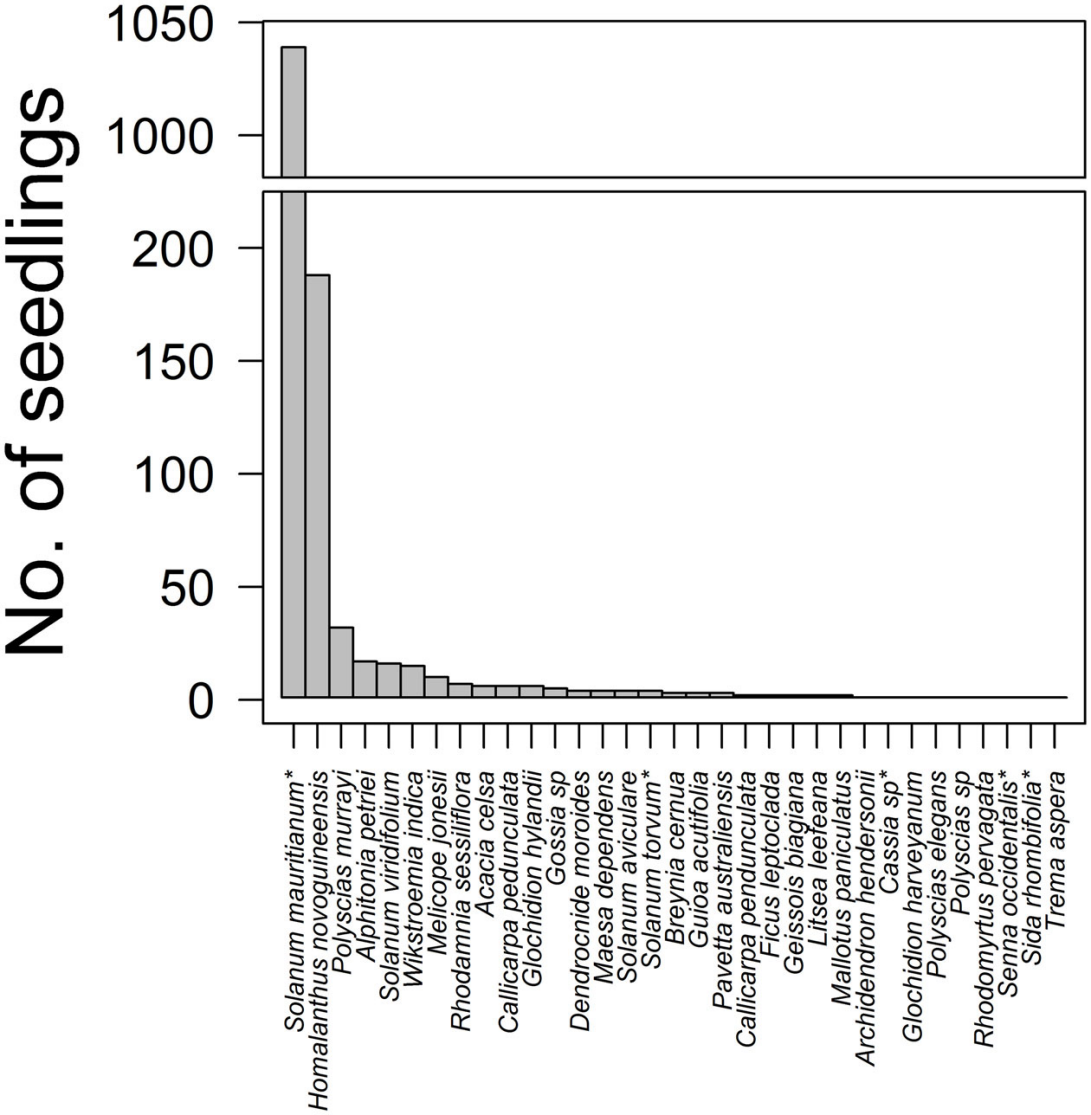
**Species frequency distribution for 1391 woody plant seedlings recorded from treated and untreated areas of disused pasture, 20 months after treatments commenced at two sites (CloudlandE, CloudlandW)**. Total sampling effort covered 4262 m^2^, in 94 strip and 40 circle surveys distributed across 2.56 ha. Stars indicate non-native species.

A total of 1391 woody plant seedlings were recorded overall from strip (*N* = 94) and circle (*N* = 40) surveys at the two Cloudland sites at 20 months (after excluding strips that intersected with pre-existing trees and shrubs). Of these, 345 were seedlings of 28 native rainforest plant species, the most common being the early successional trees *Homalanthus novoguineensis* (Euphorbiaceae, 54%), *Polyscias murrayi* (Araliaceae, 9%), *Alphitonia petriei* (Rhamnaceae, 5%), *Solanum viridifolium* (Solanaceae, 5%), and *Wikstroemia indica* (Thymelaeaceae, 4%) (Figure 1). The most commonly recruited native species varied spatially: *H. novoguineensis* was dominant at CloudlandW (70% of 253 recruits) whereas *A. petriei* dominated CloudlandE (18% of 92). Five non-native species were recorded. However, *S. mauritianum* (Solanaceae) was consistently by far the most common, comprising 99% of 1046 seedlings (Figure 1); 98% of 97 at CloudlandE and 99% of 949 at CloudlandW. In the following, we consider native and non-native seedlings separately. This enables the response of the dominant *S. mauritianum* to be effectively isolated in the analyses, allowing the response of other species to particular treatments to also be ascertained.

### Effects of grass suppression on recruitment

**Figure 2:**
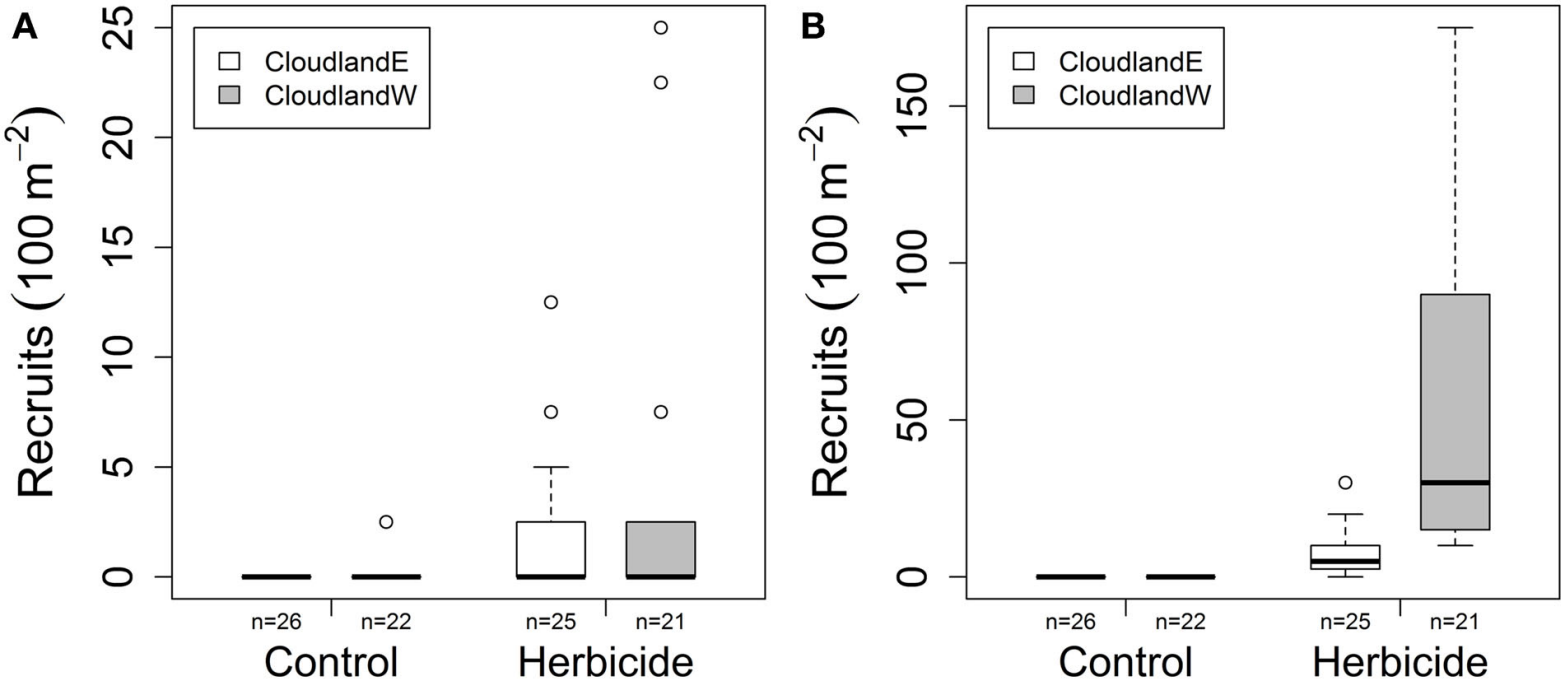
**Effect of herbicide treatment on the density of recruited woody seedlings (>10 cm tall) after 20 months at two disused pasture sites (CloudlandE, CloudlandW): (A) native and (B) non-native species**. Boxes encompass the upper and lower quartile of data with median value indicated by horizontal line. Whiskers are maximum or minimum values excluding outliers (circles) that are more than 1.5 times the upper or lower quartile.

Site and grass suppression had statistically significant and independent effects on the recruitment of woody seedlings after 20 months, both for native species (GLM site, grass suppression, interaction χ^2^ = 19.068, 146.987, 1.682; *P* < 0.001, 0.001, 0.195; Figure 2A) and non-native species (GLM site, grass suppression, interaction χ^2^ = 986.2, 2011.0, 0.0; *P* < 0.001, 0.001, 0.999; Figure 2B). Mean native seedlings recruitment increased marginally following grass suppression with slightly greater recruitment overall at CloudlandW than CloudlandE (from 0 to 1.7 seedlings per 100 m^2^ at CloudlandE; from 0.1 to 3.7 seedlings per 100 m^2^ at CloudlandW). Recruitment attributable to non-native species was also greater following grass suppression and at the CloudlandW site where mean abundance of stems reached 57.5 seedlings per 100 m^2^ (Table 1).

**Table 1:** **Effect of herbicide treatment and habitat structures on the density of recruited native and non-native seedlings at three disused pasture sites**.

**Site**	**Treatment**	**Duration (months)**	n^*^	Search type^**^	**Mean native seedling abundance (per 100 m^2^)**	**Mean non-native seedling abundance (per 100 m^2^)**
CloudlandE	Control	20	26	S	0.0	0.0
	Herbicide—Open	20	25	S	1.7	7.9
	Herbicide—Perch	20	9	C	15.0	5.3
	Herbicide—Live tree	20	7	C	66.0	13.6
CloudlandW	Control	20	22	S	0.1	0.0
	Herbicide—Open	20	21	S	3.7	57.5
	Herbicide—Perch	20	9	C	64.6	101.7
	Herbicide—Dead clump	20	10	C	84.4	177.6
	Herbicide—Live tree	20	5	C	66.9	203.8
Ringtail	Control	9	6	S	0.0	0.8
	Herbicide—Open	9	23	S	1.1	20.0
	Herbicide—Perch	9	9	C	0.9	13.3
	Herbicide—Dead clump	9	3	C	0.0	0.0
	Herbicide—Live tree	9	11	C	6.5	0.7

* n = number of strip or circle searches;

** *S = strip search of area 40 m^*2*^; C = circle search of area 12.6 m^*2*^*.

### Effects of structure on recruitment

**Figure 3:**
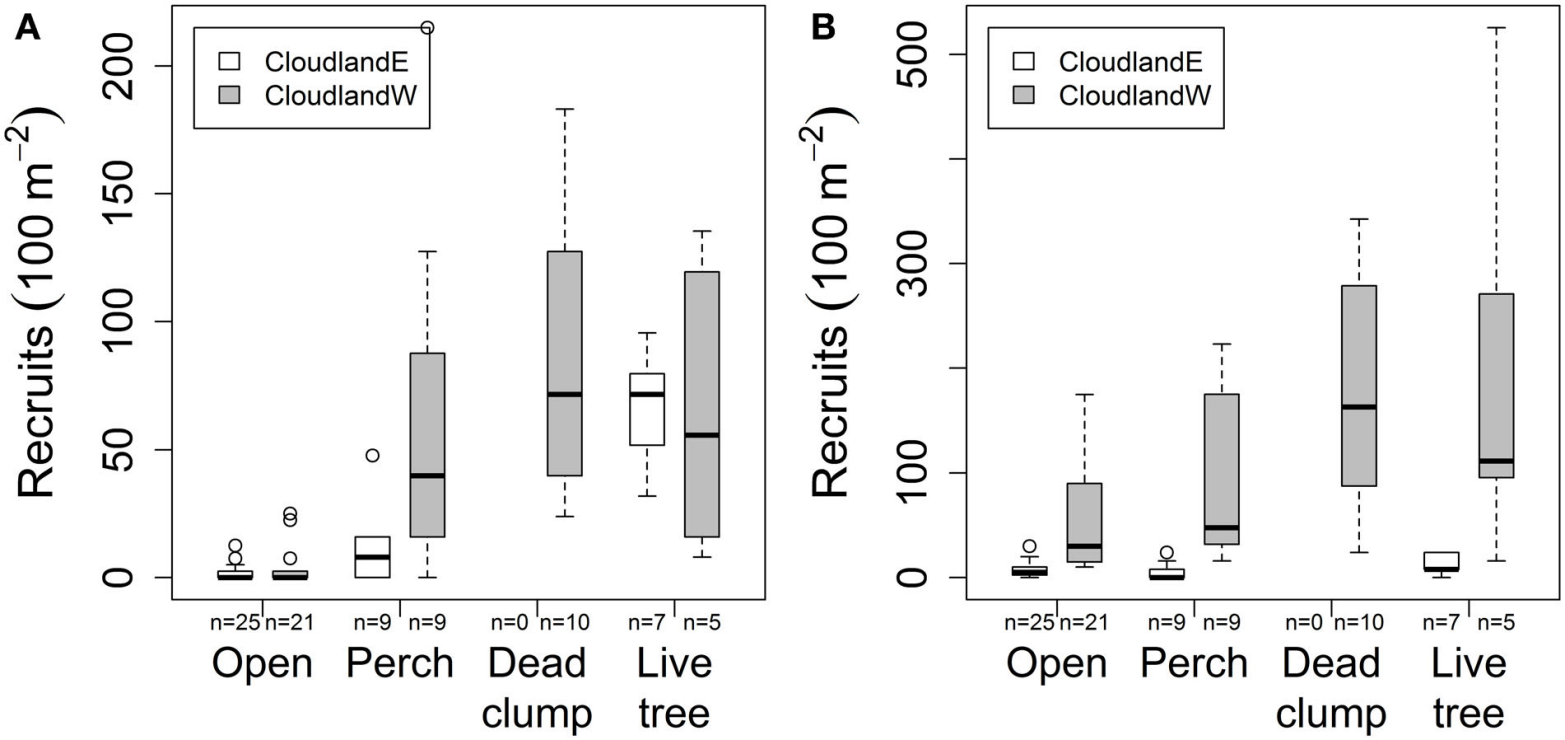
**Effect of habitat structures (perches, dead clumps, live trees) on the density of recruited woody seedlings (>10 cm tall) after 20 months at two disused pasture sites (CloudlandE, CloudlandW): (A) native and (B) non-native species**. Boxes encompass the upper and lower quartile of data with median value indicated by horizontal line. Whiskers are maximum or minimum values excluding outliers (circles) that are more than 1.5 times the upper or lower quartile.

In areas where grasses had been suppressed, native seedling recruitment at 20 months was further affected by an interaction between site and habitat structures (GLM site, structure, interaction χ^2^ = 496.61, 2716.65, 164.77; *P* < 0.001, 0.001, 0.001; Figure 3A). At CloudlandE, mean seedling recruitment was 8.8 and 38.8 times greater under perches and live trees respectively, than in open areas were only grasses had been suppressed (Table 1). At CloudlandW, the effect of perches was greater and live trees less, engendering a similar response in seedling recruitment to the two structure types (17.4 and 18.1 times greater for perches and live trees, respectively, relative to open areas; Table 1). The presence of a third structure type, dead clumps, at CloudlandW induced a larger recruitment response than either perches or live trees (22.9 times more than open areas; Table 1).

The response of non-native seedlings was governed by a different form of interaction between site and structure (GLM site, structure, interaction χ^2^ = 4345.5, 1294.4, 51.7; *P* < 0.001, 0.001, 0.001; Figure 3B). At CloudlandE, mean recruitment of non-native seedlings was marginally less under perches (0.7 times) than in open areas but was enhanced by the presence of live trees (1.7 times greater; Table 1). The effect of perches and live trees was both positive and larger at CloudlandW, where seedling recruitment was 1.8 and 3.5 times greater under the two structure types respectively than in open areas. The effect of dead clumps was similar to that of live trees, with 3.1 times more recruitment than in open areas (Table 1), although the result for live trees was highly variable (Figure 3B).

Overall, the presence of structure also had a localized effect on the proportional representation between native and non-native seedlings. At CloudlandE, abundance of native seedlings was consistently greater than non-native seedlings under all structure types despite the opposite pattern in open areas where only grasses had been suppressed (Table 1). At CloudlandW, structure also increased proportional representation of native seedlings though non-native seedlings still remained more abundant (Table 1). The 9 month data for the Cloudland sites exhibited similar patterns in response to structure as did the 20 month data though the differences were less pronounced (data not shown). The third, younger, site (Ringtail, surveyed 9 months after establishment), showed a broadly similar pattern: herbicide alone caused a small increase in native and a larger increase in non-native recruitment, whereas live trees were associated with the largest increases in native recruitment (Ringtail site; Table 1).

Across both Cloudland Works plots, the mean height of native seedlings recorded in the analyzed strips and circles after 20 months was 61 cm (range 10–250 cm, median = 23 cm, with quartiles at 12 cm and 83 cm, *N* = 338; noting that minimum measurement threshold was 10 cm and individuals >2 m were assigned a height of 2.5 m).

For native recruits, the area-specific species richness values were strongly and positively correlated with their measured densities: for strips in open areas of Control and Works plots Spearman's *r* = 0.99 at CloudlandE and 0.99 at CloudlandW (*N* = 51, 43; *P* < 0.001, 0.001 respectively); and for circles across both types of plot Spearman's *r* = 0.92 at CloudlandE and 0.66 at CloudlandW (*N* = 16, 24; *P* < 0.001, 0.001 respectively).

## Discussion

### Effects of barrier-lowering interventions on rainforest seedling recruitment

Almost no new seedlings emerged from the retired pasture Control plots over this study's 20 month period, supporting the idea that recruitment barriers are present. Repeated applications of herbicide to suppress the pasture grasses and herbs had a modest but discernable impact to enhance native seedling recruitment. This finding is broadly consistent with other regional and international studies where various interventions have been employed to alleviate ground conditions and promote the short term recovery of woody vegetation on degraded tropical land, although many of these did not differentiate between native and non-native species (Shoo and Catterall, 2013).

The recruitment of native rainforest seedlings in pasture where grasses had been suppressed was further enhanced by the presence of structural features, being greater under perches, live trees, and dead shrubs than in open areas. This is consistent with the notion that tropical forest trees can be both dispersal and establishment limited (Holl, 2012), and hence our findings add to a growing body of evidence that combined restoration interventions to simultaneously reduce multiple ecological barriers are more likely to yield tangible progress toward forest recovery than do individual techniques that target single ecological barriers (Holl et al., 2000; Posada et al., 2000; Ammondt et al., 2013). For example, in non-native grasslands of Hawaii, Brooks et al. (2009) found that broadcasting seed in conjunction with grass suppression (by herbicide application) was much more effective in enhancing the woody seedling recruitment than the summed effects of the individual techniques taken separately. Furthermore, strong correlations between species richness and abundance of natives reveal that the response of native rainforest seedlings is not just the result of one or two species but rather is underpinned by a suite of colonizing species.

Previous studies have demonstrated that perches can elevate seed rain into pasture but that this does not necessarily lead to increased seedling establishment (Holl, 1998; Shiels and Walker, 2003; Graham and Page, 2012). Our contrasting result likely stems from the fact that perches were installed in conjunction with active intervention to suppress competition from pasture grasses. However, the extent to which perches enhanced recruitment was modest when compared with the much greater recruitment beneath live trees or dead non-native shrubs. The importance of isolated trees as focal points for bird activity and seedling recruitment in pasture landscapes is well recognized (Toh et al., 1999; Manning et al., 2006). In our study, the larger effect of all trees and shrubs relative to perches is logical for two reasons. First, they had been established for a longer prior time-period, during which repeat frugivore visits could promote greater accumulation of deposited seeds. Second, the trees and shrubs had greater physical volume and architectural complexity, and potentially offered more food or shelter to attract visits by seed-dispersing birds. The dead non-native shrubs were present as a decaying skeletal framework during the 20 months of the study, and live native trees could have provided the birds with more food resources (fruit or insects) as well as shade to ameliorate local climate (thereby potentially increasing seedling germination or survival), but these factors appear relatively unimportant because the dead shrubs tended to produce greatest native seedling recruitment. This finding accords with the suggestion by Toh et al. (1999) that the identity or fruiting status of trees matter less to the activity of seed dispersing birds near forest edges than their structure and suitability as perches.

We might also expect a spatial signature in the pattern of seedling recruitment because seed rain typically decreases with increasing distance from the forest edge, with most seed rain occurring closest to the forest edge (Willson and Crome, 1989; Cubiña and Aide, 2001). However, an exploratory analysis of the effect of distance from the forest edge did not reveal a discernible pattern in seedling recruitment, which instead was highly variable and inconsistent among sites. Given this finding, together with the limited sample size for structures, and also because distance from the edge could conceivably be correlated with several factors which may independently affect recruitment in different directions (seed rain, frugivore visits, slope, exposure to forest-associated marsupial herbivores), we did not include it as a factor in the analyses presented here.

### Responses and roles of non-native woody species

**Figure 4:**
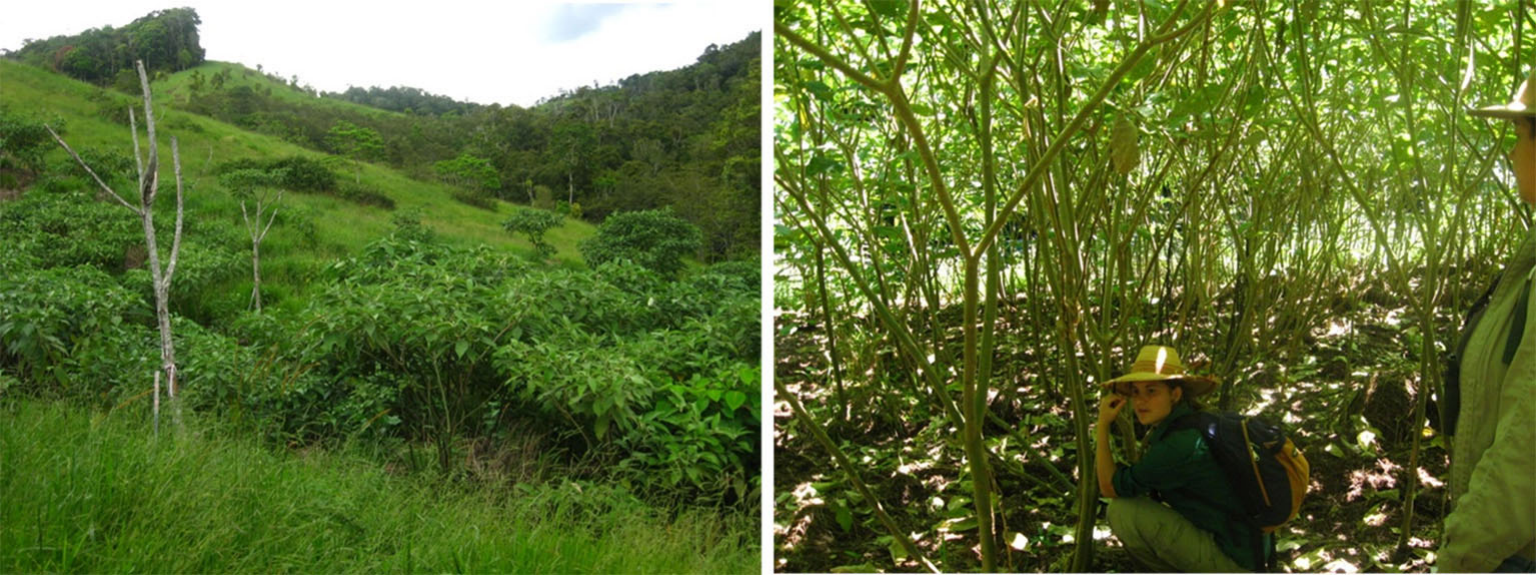
**Visual illustrations of different parts of a “Works” plot. Left panel**—14 months after initial herbicide application (viewed from outside plot). Here, bird perches can be seen above the emerging canopy of non-native wild tobacco (*Solanum mauritianum*). **Right panel**—17 months after initial herbicide application (viewed from inside plot). Here, the cover of *S. mauritianum* has shaded the ground sufficiently to suppress pasture grasses.

The most prominent short term outcome of herbicide-induced grass suppression in our study was the dramatic increase in the abundance and dominance of the non-native wild tobacco (*S. mauritianum*) which constituted the vast bulk of woody stems in open areas, and formed an extensive closed canopy 3–4 m tall over substantial parts of one experimental site (CloudlandW) within 20 months (Figure 4). Canopy dominance by a small number of light demanding tree species is not unusual in early stage succession (Guariguata and Ostertag, 2001). However, the non-native origin of the dominant species involved in this instance raises additional considerations for ongoing management.

Life history attributes of *S. mauritianum* include copious seed production and well-developed seed dormancy mechanisms, leading to high seedling recruitment from soil-stored seeds, and this species has been noted for its invasion potential (Florentine et al., 2003; Florentine and Westbrooke, 2003; Witkowski and Garner, 2008). Its relatively high soil seed storage explains why the ratio of recruit density in open areas vs. beneath habitat structures was higher for non-native woody recruits than was the case for native rainforest recruits (Table 1). Its fruit are fleshy and bird-dispersed, and hence seedling recruitment was nevertheless further enhanced by habitat structures.

This ability to colonise pasture, form a shady canopy, and quickly produce crops of fruit which are attractive to many seed dispersing frugivores (Florentine et al., 2003) may facilitate subsequent forest regeneration by greatly reducing ecological barriers of ground competition and propagule supply, much more rapidly and extensively than any native rainforest species appears capable of achieving. Indeed, Toh et al. (1999) suggested that lower strata trees such as *S. mauritianum* in the Australian subtropics likely perform an important function in providing the primary means by which taller canopy trees first enter former pasture sites. Our study's finding that native rainforest seedling recruitment was significantly enhanced beneath recently killed dead shrubs (among which *S. mauritianum* was well represented) supports the view that this species has ecological properties which facilitate forest succession. It is therefore possible that the new *S. mauritianum* stands which emerged after pasture suppression will provide positive feedback to enhance future seedling recruitment.

However, it is also possible that germination and growth of seedlings from the native seed rain falling into these stands will be suppressed by competitive interaction with the established *S. mauritianum*, necessitating future treatments to remove or thin the canopy in order to release a further rainforest seedling cohort. The notion that *S. mauritianum* may hinder recruitment of tropical rainforest pioneer and climax species stems partly from observations of an apparent absence of recruitment beneath the canopy of mature plants (Florentine et al., 2003). Shade house experiments show that concentrated aqueous leachates of *S. mauritianum* leaves inhibit the germination of lettuce seeds (*Lactuca sativa*) and can impair the early shoot and root growth of some native tropical rainforest trees (Florentine and Westbrooke, 2003). It is unknown whether these effects extrapolate to local field conditions and how they compare with the alleleopathic potential documented for some native pioneers (e.g., *Acacia melanoxylon*; González et al., 1995). In the Australian subtropics, low recruit abundances were reported beneath both *S. mauritianum* and the common native rainforest pioneer *Homalanthus nutans* which it resembles in stature (Toh et al., 1999).

The extensive recruitment of wild tobacco into retired pasture poses an important management dilemma, since it is necessary to weigh its potential benefits in reforestation against any undesirable ecological consequences of its dominance in early successional plant communities. In Australia, where suppression of invasive non-native species is a central aspect of contemporary restoration activities, routine control of *S. mauritianum* has been widely advocated in recent decades (e.g., Ward et al., 2001), although the species is not currently a declared weed under legislation and had been identified in an early ecological study as a transient successional species in forest regeneration (Williams et al., 1969). Clearly, there is a need for further research into the extent and mechanisms of the various competitive relationships that may occur between established trees and recruiting seedlings of both native and non-native woody species in an oldfield context.

More generally, there is an emerging viewpoint that while some non-native species can cause significant environmental problems, their removal may also have unforeseen consequences, and some may arguably be useful in restoration (D'Antonio and Meyerson, 2002; Reid et al., 2009). For example, in subtropical Australia, mature stands of the invasive tree *Cinnamomum camphora* in retired pasture support a high diversity of frugivorous birds that facilitate recruitment of a diverse suite of rainforest plants (Neilan et al., 2006; Kanowski et al., 2008). Particularly in moist tropical forest landscapes, some fleshy-fruited non-native tree species may thus provide opportunities for cost effective broad scale reforestation on retired agricultural land. In the restoration context, such realizations have prompted calls for non-native species to be judged on a broader understanding of factors such as their potential transience at a site and their roles in changing processes that influence the course of succession, rather than solely on their origins (D'Antonio and Meyerson, 2002; Davis et al., 2011).

Our findings likewise lead to a conclusion that evaluation of non-native pioneers' transience at sites is critical to making informed management decisions. Like many of its congeners, *Solanum mauritianum* is considered to be highly light-demanding and generally only persists and reproduces as long as the tree canopy remains relatively open (Murphy et al., 2008). This is consistent with evidence that the species declines over time during forest succession (Williams et al., 1969) and with declining light levels following closure of canopy gaps (Enright et al., 1993). In this study, many early-recruiting *S. mauritianum* individuals achieved the species' maximum height of about 4 m within less than 2 years. Most of the native species with which it co-recruited typically reach heights of at least 6 m (some considerably more), and so have the potential to overtop it. While species' growth rates vary, some *H. novoguineensis* individuals had grown as tall as, or taller than, the *S. mauritianum* within the 20 months of this study. Longer-term monitoring is needed to determine the capacity and time required for self-organized recruitment and growth of rainforest trees to shade out stands of *S. mauritianum*, and how well this development trajectory corresponds with time preferences for restoration outcomes.

### Conclusions and implications

This study illustrates the role of shifting patterns of competitive inhibition, and its interactions with ecological facilitation, during old field succession in retired agricultural land. Initially pasture grasses outcompete rainforest seedlings, but any forest trees that do establish, as well as invasive woody shrubs, can then outcompete the grass. Subsequently there are also bidirectional potential competitive processes between native and invasive woody species. Understanding of the nature of interactions between different plant functional groups, in terms of both life-form and species origin, is fundamental to these processes, and has important practical applications in the imperative to achieve rapid reforestation of large areas of degraded agricultural land in the tropics. Strategic interventions can accelerate forest recovery, if appropriately selected and timed on the basis of this knowledge. Our findings reaffirm the importance of combined intervention approaches that are designed to overcome multiple ecological barriers, in some cases simultaneously and in others sequentially. Our findings also highlight the importance of retaining or actively reinstating “islands” of woody vegetation as regeneration foci in restoration (Zahawi et al., 2013). Beneath habitat structures, especially live trees and dead shrubs, the recruited woody plant community at our study sites shifted substantially toward greater proportional representation of native seedlings. Thus, the presence of structure is likely to have important consequences for the longer term trajectory of community development.

A crucial gap in knowledge is the timeframe required for larger stature native tree species recruited into the seedling community to overtop the dominant non-native woody pasture invaders such as *S. mauritianum* and whether further interventions such as selective clearing or planting will be required to hasten this process. Our incidental field observations also suggest that the potential importance of herbivory by wildlife in mediating the recruitment of recruited seedlings in retired tropical pastures (see also Holl and Quiros-Nietzen, 1999) merits further investigation.

Empirical tests of the effectiveness of strategies other than active tree planting to simulate regeneration of tropical forest on degraded land have increased considerably over the last two decades (Shoo and Catterall, 2013). However, a major limitation to current knowledge is the short timeframes over which different interventions have been evaluated, and our study is no exception. Clearly, ongoing monitoring would be beneficial to reveal the longer-term trajectories of sites and the efficacy of alternative interventions employed to “kickstart” forest regeneration. In particular, such information would be instructive in determining whether the lower initial cost of less intense interventions considered here is offset by a longer and more expensive subsequent maintenance schedule than is typically needed after high density tree planting. Finally, this study has revealed a strong site based signature on rates of restoration progress, which means that experimental restoration projects need good landscape scale replication before the generality of findings can be fully ascertained.

### Conflict of interest statement

The authors declare that the research was conducted in the absence of any commercial or financial relationships that could be construed as a potential conflict of interest.

## Supplementary material

**Supporting Information to accompany:** Elgar et al. “Overcoming barriers to seedling regeneration during restoration on tropical pasture land and the potential value of woody weeds”

Figure S1 **Location of experimental sites in relation to each other and existing forest**. “Works” plots and “Ungrazed control” plots (80 × 80 m) are depicted by shaded and unshaded boxes respectively.

Figure S2 **“Works” plot with layout of seedling search strips and perches**.
