# Supplementary material for: Overcoming barriers to seedling regeneration during forest restoration on tropical pasture land and the potential value of woody weeds
Source: Front Plant Sci. 2014 May 20;5:200. doi: 10.3389/fpls.2014.00200 (PMC4033048; doi:10.3389/fpls.2014.00200)
Supplement: Figure S1 — Location of experimental sites in relation to each other and existing forest. “Works” plots and “Ungrazed control” plots (80 × 80 m) are depicted by shaded and unshaded boxes respectively. [file DataSheet1.DOCX]

SUPPLEMENT

**Supporting Information to accompany:** Elgar et al. ‘Overcoming barriers to seedling regeneration during restoration on tropical pasture land and the potential value of woody weeds’

**
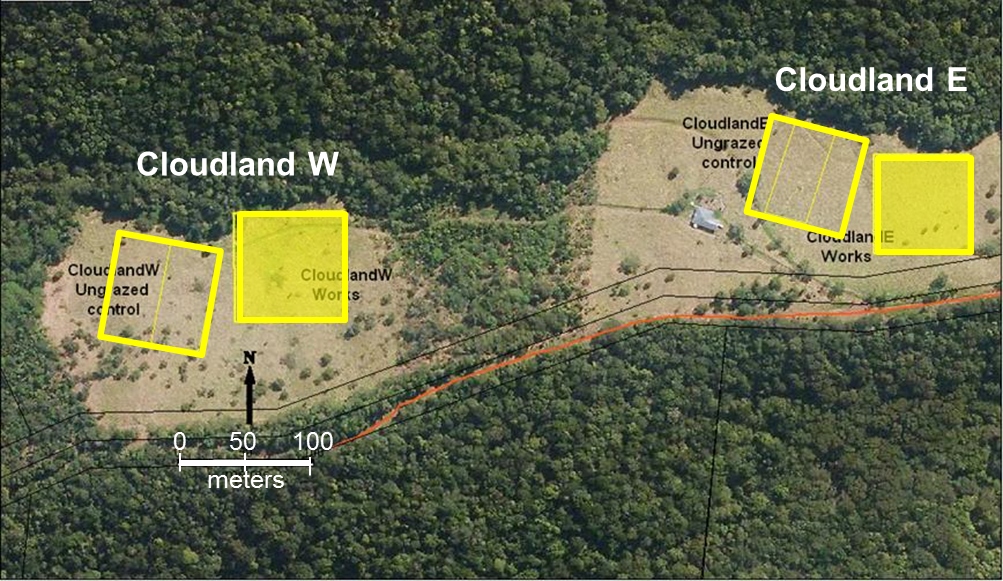
**

**FIGURE S1** Location of experimental sites in relation to each other and existing forest. ‘Works’ plots and ‘Ungrazed control’ plots (80 x 80 m) are depicted by shaded and unshaded boxes respectively.

**
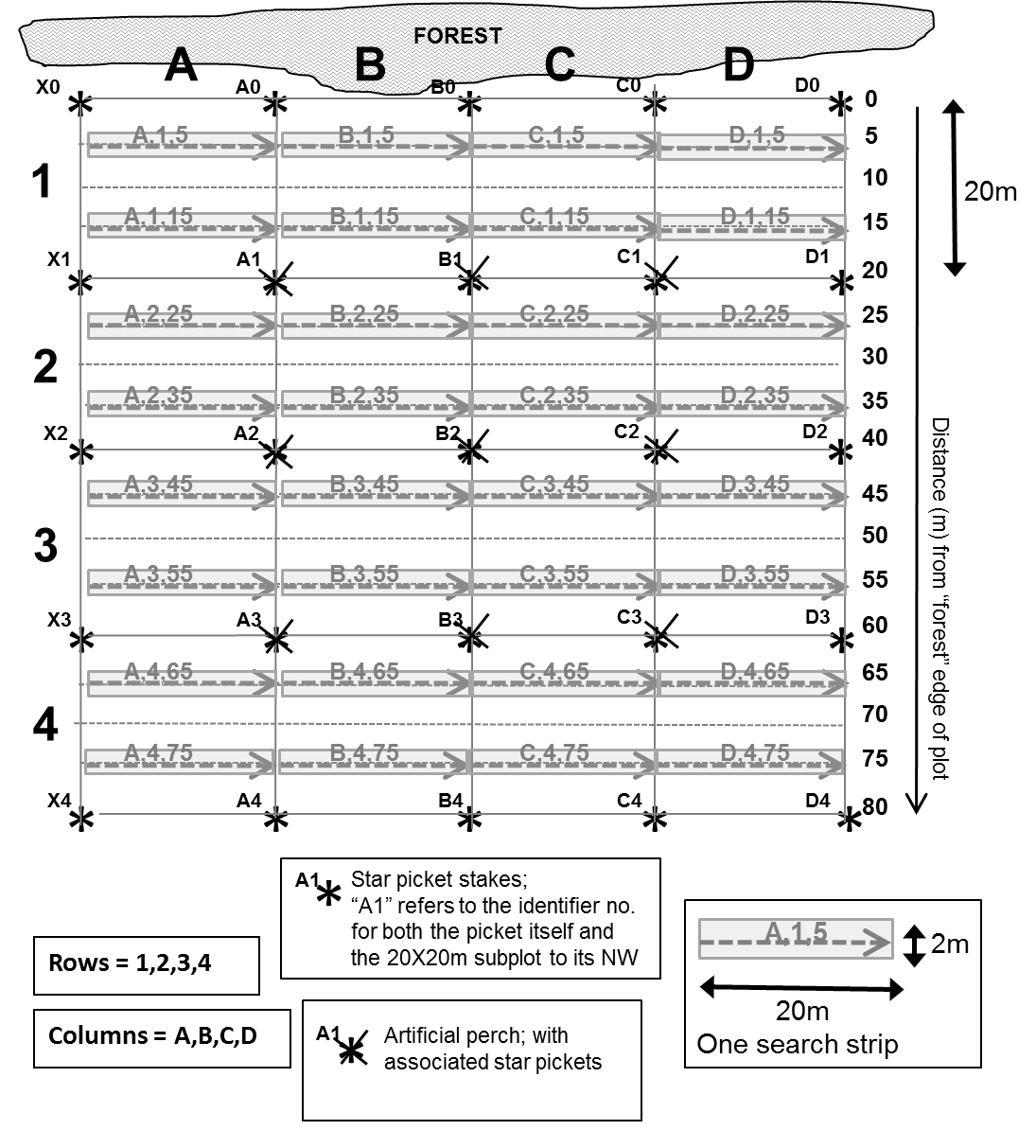
**

**FIGURE S2** ‘Works’ plot with layout of seedling search strips and perches.
